# A mechanism-oriented immersive virtual reality intervention for psychological distress and helplessness-related processes in hospitalized older adults: study protocol for a multicenter randomized controlled trial

**DOI:** 10.3389/fmedt.2026.1940940

**Published:** 2026-10-26

**Authors:** Pál Tamás Szabó, Beáta Réka Vásárhelyi, Patrícia Lajkó, Mona Stankovic, Márta Fekete, Beáta Pintérné Sosity, Samuel Chovanec, Ágnes Karolina Bakk

**Affiliations:** 1 Future Care Lab, Moholy-Nagy University of Art and Design, Budapest, Hungary; 2 Mental Health Sciences Division, Doctoral College, Semmelweis University, Budapest, Hungary; 3 Department of Rehabilitation Medicine, Semmelweis University, Budapest, Hungary; 4 Department of Geriatrics and Nursing Science, Semmelweis University, Budapest, Hungary; 5 Department of Neurosurgery, University of Szeged, Szeged, Hungary; 6 Uzsoki Hospital, Budapest, Hungary

**Keywords:** agency, digital health, hospitalized older adults, learned helplessness, perceived control, psychological distress, randomized controlled trial, virtual reality

## Abstract

**Background:**

Prolonged hospitalization in older adults may contribute to reduced autonomy, passivity, diminished perceived control, and psychological distress. Although hospitalization does not necessarily lead to learned helplessness, it may create conditions that increase vulnerability to helplessness-related processes in some patients, particularly when opportunities for self-directed action and visible action–outcome contingencies are limited. Immersive virtual reality (VR) may offer opportunities for safe, accessible, and meaningful interaction in otherwise constrained hospital environments; however, the added value of interactive, agency-supporting VR components remains insufficiently studied in hospitalized older adults.

**Objective:**

This study aims to evaluate the effectiveness of Zenctuary VR+, a mechanism-oriented, nature-based immersive VR intervention, in reducing psychological distress among hospitalized older adults compared with a passive VR control condition. Secondary aims are to explore changes in helplessness-related constructs, including self-efficacy, self-esteem, hopelessness, depressive symptoms, and learned helplessness.

**Methods and analysis:**

This is a multicenter, parallel-group, assessor-blinded randomized controlled trial conducted in inpatient hospital settings in Hungary. A total of 100 hospitalized adults aged 60 years or older will be randomly assigned in a 1:1 ratio to either the active Zenctuary VR+ intervention or a passive VR control condition. Both groups will receive six VR sessions over three weeks, delivered twice weekly using the same head-mounted display and the same nature-based virtual environments. The active intervention includes optional low-cognitive-load interactive activities designed to support agency, perceived control, and successful action, whereas the control condition involves passive observation of the same virtual environments without interactive elements. Assessments will be conducted at baseline, post-intervention, and four-week follow-up. The primary outcome is psychological distress measured by the Hospital Anxiety and Depression Scale total score. The primary analysis will use a linear mixed model to examine group-by-time effects. Secondary and mechanism-related analyses will be considered exploratory.

**Ethics and dissemination:**

The study has received national ethical and regulatory approval in Hungary from the National Centre for Public Health and Pharmacy. The trial has been registered at ClinicalTrials.gov (NCT07712549). Results will be disseminated through peer-reviewed publications, conference presentations, and project-related dissemination activities.

**Clinical Trial Registration:**

https://clinicaltrials.gov/study/NCT07712549, ClinicalTrials.gov: NCT07712549.

## 1 Introduction

Older adults undergoing prolonged hospitalization are at increased risk of functional and psychological decline. Beyond the direct effects of illness, hospitalization may restrict autonomy, reduce opportunities for self-directed activity, and place patients in a more passive role. For older adults with chronic medical conditions or other conditions requiring complex inpatient care, the hospital environment may therefore contribute not only to reduced activity, but also to diminished perceived control, lower motivation, and increased psychological distress. These effects are not universal or inevitable; rather, hospitalization may create conditions that increase vulnerability to helplessness-related processes in some patients, particularly when daily routines, mobility, treatment-related decisions, and environmental conditions are largely externally regulated. Anxiety and depressive symptoms are common in hospital settings and may interfere with rehabilitation, treatment engagement, and overall well-being (1–3).

The concept of learned helplessness provides a useful theoretical framework for interpreting these processes without assuming that hospitalization automatically produces learned helplessness. Learned helplessness refers to a psychological state in which repeated experiences of uncontrollability or non-contingency between action and outcome lead to passivity, reduced initiative, motivational decline, and depressive responses (4, 5). In hospitalized older adults, repeated experiences of limited influence over daily routines and the physical or social environment may contribute to reduced perceived control and agency. From this perspective, psychological distress in hospital settings may partly reflect constrained autonomy and limited opportunities to experience meaningful action, especially among patients exposed to prolonged dependency or uncertainty regarding recovery (6).

Interventions that restore even small forms of choice, control, and successful action may therefore be clinically relevant. Previous work on perceived control, self-efficacy, and autonomy in older adults suggests that structured opportunities for choice and personal influence can support engagement and well-being (7–10). This is particularly important in clinical contexts where patients may experience dependency, functional limitations, or uncertainty regarding recovery. Accordingly, interventions for hospitalized older adults should not only aim to reduce symptoms, but also create conditions in which patients can experience competence, agency, and meaningful engagement.

Virtual reality (VR) offers a promising approach for this purpose. VR can provide access to safe, engaging, and controllable environments even when physical mobility and access to natural surroundings are limited (11–13). Nature-based VR environments may support emotional regulation, relaxation, and positive affect, while interactive VR systems can additionally provide opportunities for exploration, choice, and environmental influence (14–17). However, the potential benefits of VR are not automatic. Older and clinically vulnerable users may experience uncertainty, cognitive overload, anxiety, or discomfort during initial encounters with immersive technology. For this reason, VR interventions designed for hospitalized older adults require careful attention to usability, onboarding, safety, and cognitive load (18–22).

Zenctuary VR+ is a nature-based VR intervention developed for older adults in clinical settings. It builds on the earlier Zenctuary VR concept (23–25) and extends it into a more structured intervention designed to provide accessible, meaningful, low-cognitive-load interactions within a safe virtual environment (15, 18, 20). Its core design rationale is mechanism-oriented: rather than offering passive exposure to pleasant virtual scenery alone, Zenctuary VR+ aims to provide repeated opportunities for successful action, perceived control, and agency. Participants can engage in simple interaction tasks, navigate between virtual locations, and influence elements of the environment while remaining seated and supported by trained personnel. These features are hypothesized to address helplessness-related processes that may emerge in response to prolonged dependency, reduced autonomy, and limited opportunities for self-directed action during hospitalization.

The hypothesized mechanism is based on repeated, low-demand opportunities for self-initiated action and visible action–outcome contingency. Such experiences may support perceived control, agency-related experience, and self-efficacy, thereby addressing psychological processes theoretically related to helplessness and passivity.

The present study is a multicenter randomized controlled trial evaluating the effectiveness of Zenctuary VR+ among hospitalized older adults. The trial compares an active, interactive VR intervention with a passive VR control condition using the same virtual environments but without interactive elements. This design allows the study to examine whether the interactive Zenctuary VR+ condition provides additional benefit beyond immersion in a virtual natural environment alone (26, 27).

The primary objective is to determine whether active Zenctuary VR+ reduces psychological distress, measured by the HADS total score, compared with passive VR exposure. Secondary objectives are to explore changes in self-efficacy, self-esteem, hopelessness, depressive symptoms, and learned helplessness. We hypothesize that the active intervention will produce greater reductions in psychological distress than the passive control condition. Agency, perceived control, and action–outcome contingency are conceptualized as hypothesized proximal experiential mechanisms and are not directly assessed using dedicated quantitative measures in the present trial. These constructs will be explored qualitatively, while the quantitative secondary measures assess broader helplessness-related psychological constructs. Mechanism-related quantitative analyses will remain exploratory and will not be interpreted as establishing causal mediation.

The conceptual model underlying the trial is shown in Figure 1. The model illustrates two parallel pathways through which older age, chronic illness, multimorbidity, functional limitation, and prolonged hospitalization may contribute to psychological distress. The environmental deprivation/stress-restoration pathway reflects reduced access to restorative environments and meaningful sensory stimulation within a medicalized and externally regulated hospital context. The control/agency deprivation pathway reflects reduced autonomy, fewer opportunities to exercise personal control, reduced perceived control and agency, and repeated experiences of limited action–outcome contingency, which may contribute to helplessness-related beliefs, reduced self-efficacy, increased hopelessness, and depressive symptoms. These pathways provide the theoretical background for the intervention and its hypothesized mechanism of action.

**Figure 1:**
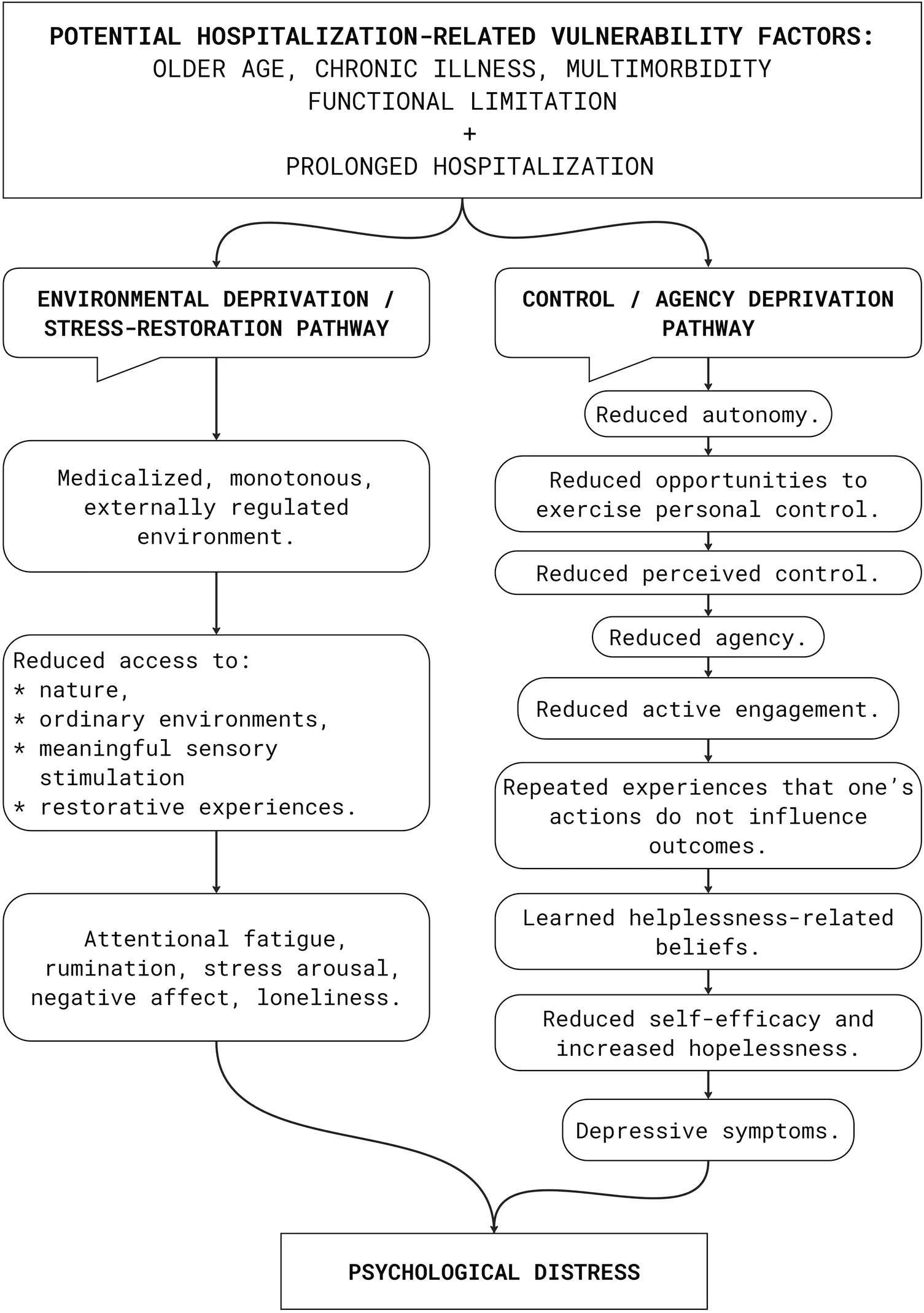
Conceptual model of hospitalization-related vulnerability and pathways to psychological distress.

## 2 Materials, methods and analysis

### 2.1 Study design

This study is a multicenter, parallel-group, randomized controlled trial designed to evaluate the effectiveness of a mechanism-oriented, nature-based immersive virtual reality (VR) intervention (Zenctuary VR+) in reducing psychological distress among hospitalized older adults. Participants will be randomly allocated in a 1:1 ratio to either an active VR intervention group or a passive VR control group.

The trial will employ an assessor-blinded design. Due to the nature of the intervention, participants and intervention operators cannot be blinded to group allocation.

The active intervention consists of an interactive nature-based VR experience designed to promote agency, perceived control, and engagement through low-cognitive-load activities. The control condition involves exposure to the same virtual environments without interactive elements, allowing participants to passively observe the virtual scenery.

The study follows a repeated-measures design with assessments conducted at baseline (T0), immediately following the intervention period (T1), and at a four-week follow-up assessment (T2). The intervention schedule and participant timeline are described below.

This protocol was prepared in accordance with the SPIRIT 2025 Statement for randomized trial protocols (28, 29). A completed SPIRIT checklist mapping the relevant protocol items to the corresponding manuscript sections is provided as Supplementary Table S1.

### 2.2 Study context

#### 2.2.1 Study setting

This multicenter study will be conducted in inpatient hospital settings in Hungary. Participant recruitment and intervention delivery will take place at collaborating clinical sites providing care for older adults with chronic medical conditions or other conditions requiring complex inpatient care. The participating sites include the Department of Rehabilitation Medicine and the Department of Geriatrics and Nursing Science at Semmelweis University, and the Department of Neurosurgery at the University of Szeged.

Participating sites are inpatient clinical centers with the capacity to support recruitment, informed consent procedures, intervention delivery, safety monitoring, and follow-up data collection. Participants will be recruited from inpatient wards in collaboration with treating physicians and clinical staff.

All study procedures, including screening, informed consent, intervention delivery, outcome assessment, and safety monitoring, will be performed on-site within the participating institutions. The intervention will be delivered individually using a head-mounted virtual reality display (Meta Quest 3) under the supervision of trained study personnel. Intervention operators will be required to complete study-specific training before delivering VR sessions.

All participating sites will follow the same study manual, safety procedures, data collection forms, and reporting requirements. The study will therefore be implemented according to a standardized protocol across all centers to ensure consistency in recruitment, intervention delivery, data collection, follow-up procedures, and safety reporting.

#### 2.2.2 Study duration

The study is planned to commence in November 2026 and conclude in December 2027. Participant recruitment, intervention delivery, follow-up assessments, and data collection will be conducted throughout this period.

Each participant will take part in the study for approximately seven weeks (a three-week intervention period plus a four-week follow-up period after the final VR session). Following eligibility screening and baseline assessment (T0), participants will undergo a three-week intervention period consisting of six VR sessions delivered twice weekly. Post-intervention assessments will be conducted immediately after completion of the intervention period (T1). A follow-up assessment (T2) will be performed four weeks after the final intervention session to evaluate the persistence of intervention effects.

Outcome measures will be collected at three predefined time points: baseline (T0), post-intervention (T1), and four-week follow-up (T2). To reduce participant burden, particularly among hospitalized older adults with limited physical or cognitive endurance, questionnaire administration may be divided across two separate assessment sessions occurring within a maximum interval of 48 h.

#### 2.2.3 Patient and public involvement

The development of Zenctuary VR+ was informed by participatory and practice-based development activities involving older adults, clinical stakeholders, and interdisciplinary design and research team members. Feedback from preparatory workshops, usability testing, and clinical consultations informed the design of the virtual environments, interaction logic, onboarding procedures, safety considerations, and operator-assisted accessibility features.

Patient and user input contributed particularly to the simplification of interaction demands, the development of preparatory onboarding procedures, the refinement of safety communication, and the emphasis on seated, low-cognitive-load participation. Although patients and members of the public will not be involved in randomization, outcome assessment, or statistical analysis, their earlier input shaped the intervention design and implementation procedures. Findings from the trial will be disseminated through scientific publications, conference presentations, and project-related dissemination activities.

### 2.3 Participants and eligibility criteria

Participants will be recruited from inpatient hospital wards across the participating study centers. The target population comprises hospitalized adults aged 60 years or older with at least one chronic medical condition and/or a medical condition requiring complex inpatient care, who are receiving care in geriatric, rehabilitation, neurological, or related inpatient settings and are expected to require prolonged hospitalization. For the purposes of this trial, complex inpatient care refers to ongoing hospital-based management requiring coordinated multidisciplinary medical, nursing, rehabilitative, supportive, or other specialist care, irrespective of whether the underlying condition is formally classified as chronic. For the purposes of this trial, prolonged hospitalization is operationalized prospectively as an expected inpatient stay of at least four weeks following enrollment. The intervention is intended as a supportive and potentially preventive adjunct during hospitalization; therefore, eligibility is not restricted to participants with elevated psychological distress, reduced perceived control, or other predefined helplessness-related symptoms. Eligibility will be determined through screening against predefined inclusion and exclusion criteria in consultation with the treating clinical team.

#### 2.3.1 Inclusion criteria

Participants will be eligible for inclusion if they meet all of the following criteria:

- Aged 60 years or older;
- Diagnosed with at least one chronic medical condition and/or a medical condition requiring complex inpatient care as defined above;
- Currently receiving inpatient hospital care;
- Expected to remain hospitalized for at least four weeks following enrollment;
- Physically able to remain safely seated or semi-seated and tolerate use of the head-mounted virtual reality device for the planned session duration, with operator assistance where required, as determined by the treating clinical team;
- Able to provide written informed consent;
- Able to understand simple study instructions and communicate responses sufficiently to complete the study assessments and participate in the intervention procedures.

Potential participants will be screened in consultation with their treating physician to ensure that participation is clinically appropriate and does not interfere with standard medical care.

#### 2.3.2 Exclusion criteria

Participants meeting any of the following criteria will be excluded from the study:

- A Montreal Cognitive Assessment (MoCA) score below 18 after application of the standard education adjustment (+1 point for participants with 12 years or fewer of formal education); this threshold is used as an operational eligibility criterion to exclude cognitive impairment likely to interfere with informed participation, completion of study assessments, or use of the VR intervention, rather than as a diagnostic cutoff for cognitive impairment;
- Impaired consciousness or reduced alertness, acute delirium, or fluctuating confusion at the time of screening that would prevent meaningful participation or valid informed consent;
- A current or previous diagnosis of a psychotic disorder;
- Current substance use disorder that would interfere with participation in the study procedures;
- Current treatment with antipsychotic medication;
- Untreated epilepsy;
- Severe vestibular dysfunction or recurrent vertigo;
- Any medical condition judged by the treating physician to make participation unsafe or inappropriate.

Clinical suitability, alertness, and the presence of acute or fluctuating confusion will be assessed during preliminary screening in consultation with the treating clinical team. Individuals with suspected or clinically identified delirium will not undergo study enrollment while the condition is present. Capacity to provide informed consent will be assessed by trained study personnel based on the participant's ability to understand the purpose and main procedures of the study, appreciate the voluntary nature of participation and the right to withdraw, and communicate a clear decision. If decision-making capacity cannot be established, the individual will not be enrolled.

The exclusion of participants with a current or previous diagnosis of a psychotic disorder or current antipsychotic treatment represents a conservative, protocol-defined eligibility criterion established *a priori* by the multidisciplinary clinical research team, including psychologists with experience in the clinical use of VR. Given the medically vulnerable older inpatient population and the initial clinical evaluation of Zenctuary VR+, the team considered a conservative approach to psychiatric eligibility appropriate to support participant safety, clinical interpretability, and a more homogeneous study population. These criteria are not intended to imply that immersive VR is generally contraindicated in individuals with psychotic disorders or in those receiving antipsychotic medication.

### 2.3 Recruitment

Participants will be recruited from inpatient wards of the participating clinical centers. Potential participants will be identified through collaboration with treating physicians, nurses, psychologists, and other members of the clinical care team. Clinical staff will conduct an initial screening based on the study eligibility criteria and will inform potentially eligible patients about the study.

In addition to clinician referral, passive recruitment strategies, including study information leaflets and posters displayed within participating wards, may be used to increase awareness of the study. Family members and caregivers may receive information about the study upon request.

Individuals identified as potentially eligible will receive both verbal and written information regarding the study objectives, procedures, potential benefits, and risks. Adequate time will be provided for consideration and questions before participation is requested. Written informed consent will be obtained by trained study personnel who are not directly involved in the participant's routine clinical care. Enrollment will occur only after written informed consent has been obtained.

After written informed consent has been obtained, participants will undergo study-specific eligibility assessments, including cognitive screening with the Montreal Cognitive Assessment (MoCA). Participants who meet all eligibility criteria will then proceed to baseline assessment before randomization.

Participation in the study is entirely voluntary. Declining participation or withdrawing from the study at any time will not affect the participant's medical care, treatment, or relationship with the healthcare institution. Participants who discontinue VR sessions but do not withdraw consent will be asked to complete the remaining outcome assessments whenever feasible. To ensure consistency across study sites, all participating centers will follow a standardized recruitment and informed consent procedure.

Participants who consent to study participation may additionally provide separate consent for follow-up contact after hospital discharge, including telephone-based follow-up assessments when in-person assessment is not feasible.

### 2.4 Training

All study personnel involved in participant recruitment, intervention delivery, outcome assessment, and data management will receive standardized study-specific training before the start of participant enrollment.

Personnel responsible for intervention delivery will be trained in the operation of the Zenctuary VR+ system, participant onboarding procedures, use of the operator interface, safety monitoring, and adverse event management. Training will include both theoretical instruction and supervised practical sessions to ensure consistent intervention delivery across study sites. Training will include the standardized least-assistance procedure described in the Study intervention section, including appropriate use and documentation of operator support.

Outcome assessors will receive separate training on study procedures, questionnaire administration, and data collection protocols.

Training will also cover procedures for documenting protocol deviations, session interruptions, missed sessions, adverse events, implementation challenges, and participant withdrawals. Intervention fidelity will be supported through standardized onboarding materials, operator instructions, session logs, and regular coordination meetings across participating sites.

A standardized study manual and operating procedures will be provided to all participating centers. Prior to participant enrollment, all study personnel will be required to demonstrate competency in their assigned study procedures. Regular meetings will be conducted throughout the study period to review protocol adherence, address procedural issues, and ensure consistency across sites. Any protocol deviations, adverse events, or implementation challenges will be documented and reviewed by the study coordination team.

### 2.5 Randomization and allocation concealment

Participants will be randomly assigned in a 1:1 ratio to either the active VR intervention group or the passive VR control group. A computer-generated block randomization sequence will be prepared by an independent researcher who is not involved in participant recruitment, intervention delivery, outcome assessment, or data analysis.

The randomization sequence will use concealed variable block sizes and will be stratified by study site to support balance across participating centers. The final randomization list will be prepared before the start of recruitment and will be stored in a secure location inaccessible to personnel involved in recruitment, eligibility assessment, informed consent, baseline data collection, outcome assessment, or data analysis.

Allocation concealment will be maintained through centralized computer-generated randomization performed only after participant enrollment and completion of baseline assessment. The randomization sequence will be inaccessible to personnel involved in participant recruitment, eligibility assessment, informed consent procedures, and baseline data collection (30).

Participants will be enrolled by trained study personnel. Randomization will be initiated only after eligibility has been confirmed, written informed consent has been obtained, and baseline assessment has been completed. The participant will then be assigned to one of the two study groups according to the pre-generated randomization sequence.

Group allocation will be communicated only to the personnel responsible for intervention scheduling and delivery.

### 2.6 Blinding

Due to the nature of the intervention, participants and intervention operators cannot be blinded to treatment allocation. However, outcome assessments will be conducted by trained assessors who are not involved in intervention delivery and remain unaware of participants' group assignment. Role separation between intervention operators and outcome assessors will be implemented at each participating site. Whenever possible, outcome assessments will be performed by personnel who do not deliver VR sessions. If staffing constraints at a site limit full personnel separation, the same staff member will not both deliver the intervention and conduct outcome assessments for the same participant. Assessment scheduling and documentation procedures will be used to preserve assessor blinding throughout the trial. Participants will be instructed not to disclose their allocation during assessment procedures. Any accidental disclosure of group allocation to an outcome assessor will be documented as a protocol deviation. As participants and intervention operators are not blinded, emergency unblinding procedures are not applicable. If feasible, the primary statistical analysis will be conducted using coded group labels (31).

### 2.7 Measurements and assessments

Baseline demographic and clinical data will be collected to characterize the study population, support eligibility assessment, ensure safe VR participation, and support interpretation of trial outcomes. Demographic data will include age and sex. Clinical data will include study site, inpatient care setting, primary clinical condition or diagnostic category, relevant chronic medical conditions, current hospitalization status, expected length of inpatient stay, and clinically relevant information related to safe VR participation, including cognitive screening result, mobility or positioning limitations, history of epilepsy or severe vestibular symptoms, current antipsychotic medication use, and other contraindications defined in the eligibility criteria. Only data necessary for eligibility assessment, participant characterization, safety monitoring, and outcome interpretation will be collected. No full medical records, laboratory results, imaging data, or detailed treatment records will be collected for research purposes unless directly required for eligibility assessment, safety monitoring, or adverse event documentation.

Outcome assessments will be conducted at three predefined time points: baseline (T0), immediately following completion of the intervention period (T1), and four weeks after the end of the intervention (T2). After written informed consent and before baseline assessment, cognitive screening will be performed using the validated Hungarian-language version of the Montreal Cognitive Assessment (MoCA) (32, 33). In accordance with standard MoCA scoring procedures, one point will be added for participants with 12 years or fewer of formal education. A score below 18 will be used as a pragmatic, prespecified operational eligibility threshold selected to exclude participants with more pronounced cognitive impairment that could interfere with informed participation, completion of study assessments, or meaningful engagement with the VR intervention; it will not be interpreted as a diagnostic threshold for cognitive impairment.

To minimize assessment burden in this hospitalized older adult population, questionnaire administration may be divided across two separate assessment sessions when required. In such cases, the interval between sessions will not exceed 48 h.

At baseline (T0), participants will complete the Hospital Anxiety and Depression Scale (HADS), Rosenberg Self-Esteem Scale (RSES), Generalized Self-Efficacy Scale (GSES), Geriatric Depression Scale (GDS), Beck Hopelessness Scale (BHS), and the Learned Helplessness Scale (LHS) (34–40). Post-intervention assessments (T1) will include the HADS, RSES, GSES, GDS, BHS, and LHS. Follow-up assessments (T2) will include the HADS, RSES, and GSES.

Whenever possible, follow-up assessments will be conducted in person. If a participant has been discharged before the follow-up assessment, data collection may be completed through a structured telephone interview, provided that separate consent for follow-up contact has been obtained.

#### 2.7.1 Primary outcome

The primary outcome of the study is psychological distress, measured using the Hospital Anxiety and Depression Scale (HADS) (34). The HADS is a widely used self-report instrument designed to assess symptoms of anxiety and depression in medical populations (35). It consists of 14 items divided into two subscales measuring anxiety (HADS-A) and depression (HADS-D). The HADS total score was selected as the primary outcome because the intervention is intended to reduce overall psychological distress rather than anxiety or depressive symptoms alone. The HADS-A and HADS-D subscales will be examined separately as exploratory outcomes to describe whether any intervention-related change is more pronounced in anxiety-related or depression-related symptoms.

The primary analysis will evaluate changes in HADS total score over time and compare these changes between the active VR intervention group and the passive VR control group. Assessments will be conducted at baseline (T0), immediately following completion of the intervention period (T1), and at four-week follow-up (T2).

The primary hypothesis is that participants receiving the active Zenctuary VR+ intervention will demonstrate a greater reduction in psychological distress compared with participants allocated to the passive VR control condition.

#### 2.7.2 Secondary outcomes

Secondary outcomes assess broader psychological constructs theoretically related to learned helplessness and adaptation to hospitalization. They are not direct quantitative measures of the hypothesized proximal mechanisms of agency, perceived control, or action–outcome contingency.

Self-esteem will be assessed using the Rosenberg Self-Esteem Scale (RSES). General self-efficacy will be measured using the General Self-Efficacy Scale (GSES). Hopelessness will be assessed using the Beck Hopelessness Scale (BHS). Depressive symptoms will be measured using the Geriatric Depression Scale (GDS), which is included as a secondary, geriatric-specific measure of depressive symptomatology. Whereas the HADS total score serves as the primary measure of overall psychological distress, the GDS will provide additional information on depressive symptoms in this older inpatient population.

Learned helplessness will be assessed using the Learned Helplessness Scale (LHS). At the time of protocol preparation, no fully validated Hungarian version of the LHS was available. Therefore, LHS scores are planned to be interpreted as exploratory and mechanism-related rather than as primary evidence of intervention effectiveness.

Secondary outcome measures will be collected at baseline (T0) and post-intervention (T1). Selected measures, including self-esteem and self-efficacy, will also be collected at follow-up (T2) to explore the persistence of intervention-related effects.

Brief semi-structured post-session interviews will be conducted by the trained session operator immediately after each VR session. Where separate consent for audio recording has been provided, interviews will be audio-recorded and transcribed for qualitative analysis. Where audio recording is unavailable or the participant does not provide consent for recording, the operator will complete a structured written summary immediately after the interview. Transcripts of audio-recorded interviews and operator-written summaries will not be treated as equivalent data sources. Verbatim transcripts will constitute the primary source for detailed thematic analysis, whereas structured written summaries will be used as supplementary contextual data and will be identified separately during analysis.

Operators will also complete standardized session notes documenting stations visited and their order, whether participant-initiated navigation or interaction occurred, which predefined interactive activities were engaged with, assistance required, interruptions, behavioral or emotional reactions, tolerability issues, and procedural adaptations. These observational notes will be distinguished from participant interview data during analysis.

After the sixth VR session, a short final semi-structured interview will explore the participant's overall experience across the intervention period, perceived changes over time, and any future-oriented reflections elicited by the VR sessions. Where separate consent for audio recording has been provided, the final interview will be audio-recorded and transcribed; otherwise, a structured written summary will be completed. These interviews will be treated as qualitative contextual data rather than standardized outcome measures.

The participant flow and randomized trial schema are shown in Figure 2. The detailed schedule of enrollment, intervention delivery, assessments, session-level monitoring, and follow-up is provided in Table 1.

**Figure 2:**
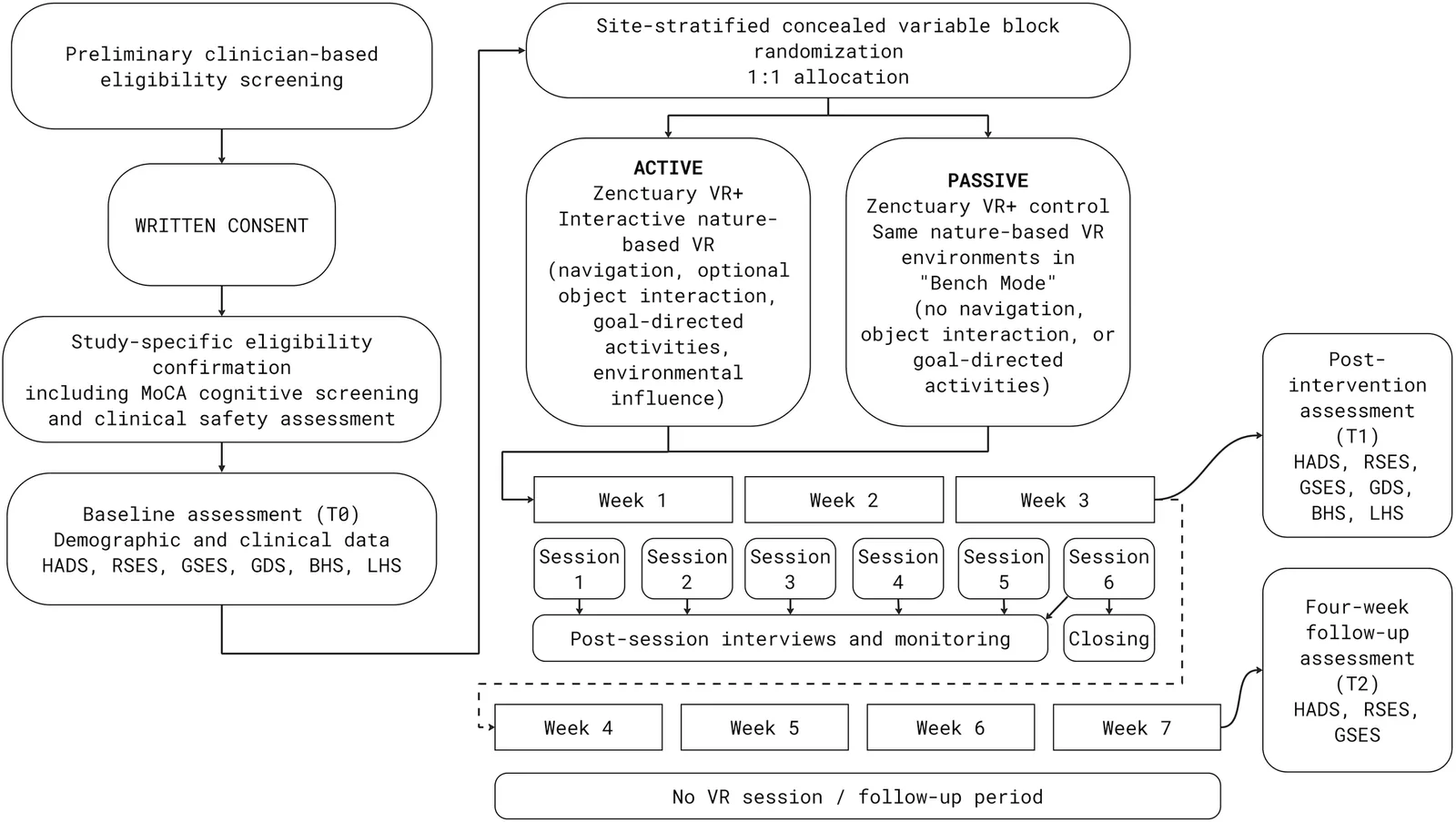
Participant flow and randomized trial schema. Potentially eligible participants will be identified through preliminary clinician-based screening before written informed consent. After consent, study-specific eligibility will be confirmed, including MoCA cognitive screening and clinical safety assessment, followed by baseline assessment (T0). Eligible participants will be randomized in a 1:1 ratio using site-stratified concealed variable block randomization to either active Zenctuary VR+ or passive VR control. Both groups will receive six VR sessions over three weeks with post-session interviews and monitoring after each session and a closing procedure after Session 6. Post-intervention assessment (T1) will be conducted after the intervention period, followed by a four-week no-VR follow-up period and follow-up assessment (T2).

**Table 1:** Schedule of enrollment, intervention, and assessments.

Study procedure	Screening/enrollment	Baseline T0	Week 1	Week 2	Week 3	Post-intervention T1	Follow-up T2
Preliminary eligibility screening	X						
Informed consent	X						
MoCA cognitive screening	X						
Demographic and clinical data		X					
HADS		X				X	X
RSES		X				X	X
GSES		X				X	X
GDS		X				X	
BHS		X				X	
LHS		X				X	
Randomization		X					
VR sessions			Sessions 1–2	Sessions 3–4	Sessions 5–6		
Session log			X	X	X		
Adverse event monitoring			X	X	X	X	
Semi-structured post-session interview			X	X	X		
Final semi-structured interview after Session 6 (closing)					X		
Withdrawal/discontinuation documentation	X	X	X	X	X	X	X

### 2.8 Study intervention

Participants allocated to the intervention group will receive the Zenctuary VR+ intervention, a nature-based immersive virtual reality program specifically designed for hospitalized older adults. The intervention was developed through a multi-phase preparatory process including participatory co-design activities, iterative prototype refinement, and formative usability work conducted prior to the present randomized controlled trial.

The intervention will be delivered using a Meta Quest 3 head-mounted display. Participants will remain seated throughout the sessions. All sessions will be conducted individually under the supervision of a trained operator.

Zenctuary VR+ was developed in Unity 6000.0.61F1 using the Universal Render Pipeline and Meta Quest Integration, with Android as the target platform. The participant-facing application runs standalone on the Meta Quest 3 headset. A separate operator-facing application communicates with the headset over a local wireless network and provides real-time monitoring and intervention support.

The intervention comprises six distinct nature-based virtual environments: a lookout, a lakeside, a riverside, a meadow, a forest, and a garden with a pavilion. Each environment contains five or six predefined stations that combine environmental observation, navigation, and optional low-cognitive-load interactions. Participants can move between stations using a simplified gaze-assisted teleportation system and interact with virtual objects through controller-based interactions.

The intervention comprises six distinct nature-based scenes, each containing five or six predefined stations that combine environmental observation, navigation, and optional low-cognitive-load interactions. The first intervention session follows the same predefined scene for all participants to provide a standardized initial experience. From the second session onward, the order of the remaining scenes may be selected according to participant preference.

The intervention incorporates a range of simple activities designed to be accessible to older adults with varying levels of physical and cognitive functioning. These activities include, but are not limited to, fruit picking, feeding virtual animals, watering plants, collecting objects, environmental exploration, and other goal-directed interactions requiring minimal motor precision. The activities were intentionally designed to promote successful engagement with simple goal-directed activities while minimizing cognitive burden and frustration, in line with previous work on VR usability and design considerations for older adults (18, 20, 41).

Within each scene, participants may choose, repeat, or discontinue available activities. No minimum number of interactions is required; some activities have a finite endpoint, whereas others are open-ended and repeatable.

The interactive elements are intended to provide opportunities for self-initiated action with visible environmental consequences. In line with the mechanism described in the Introduction, these experiences are hypothesized to support perceived control, agency-related experience, and mastery-like experience while maintaining low cognitive and motor demands. Repeated experiences of such successful, self-initiated action are hypothesized to support mastery experiences and strengthen self-efficacy (6, 7, 9, 10).

To support safe and controlled intervention delivery, the Zenctuary VR+ system combines a participant-facing immersive virtual environment with a dedicated operator-facing interface for real-time monitoring and support (Figures 3A,B). The operator interface allows real-time monitoring of participant activity and, when necessary, provides assistance with navigation or interaction. Assistance will follow a standardized least-assistance approach. Operators will first use brief verbal prompts, clarification, or reassurance and will provide direct navigation or interaction support only when required for safety, accessibility, technical reasons, or task comprehension. This approach is intended to support participation while minimizing unnecessary replacement of participant-initiated action.

**Figure 3:**
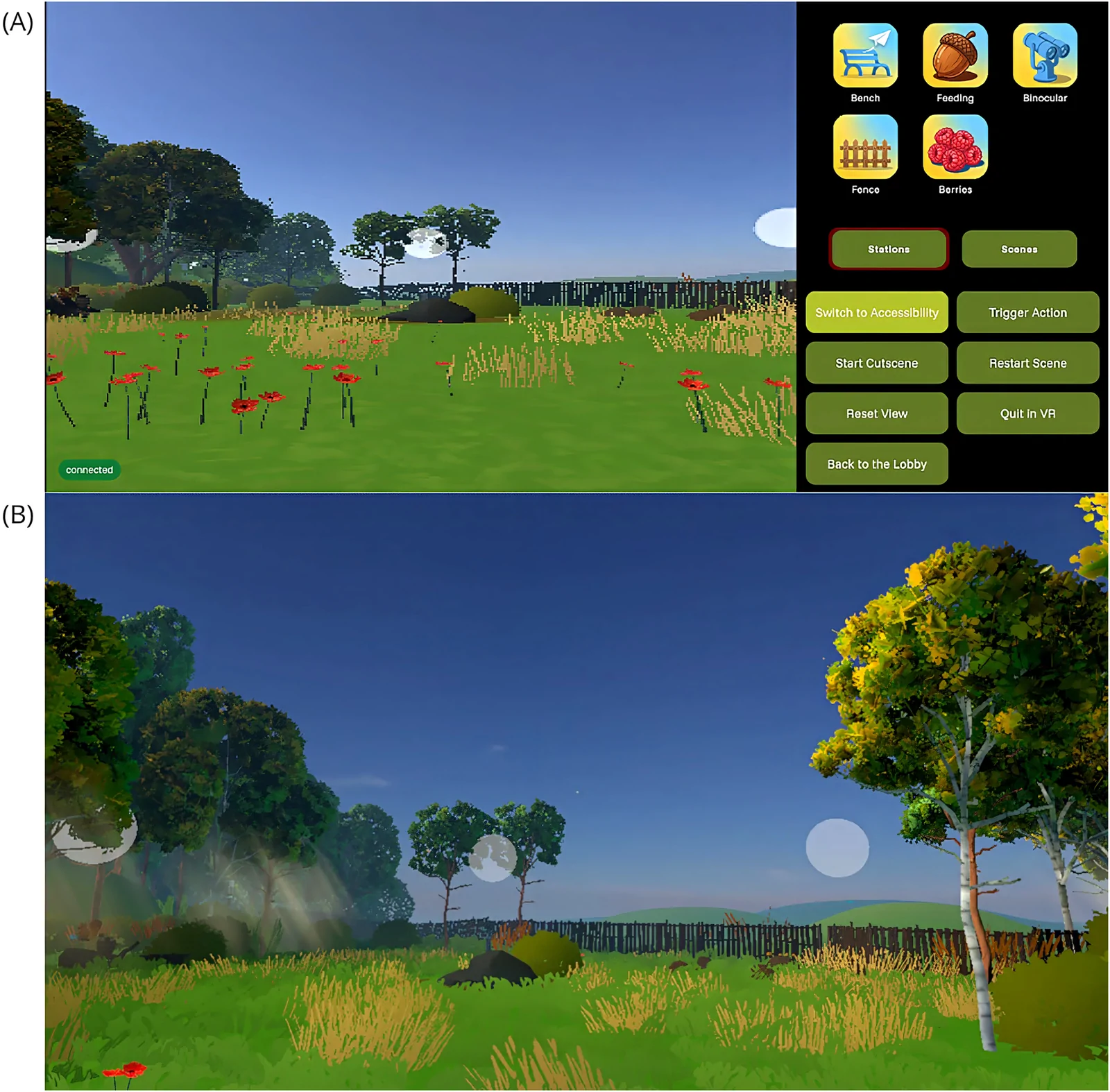
Operator-facing interface **(A)** and participant-facing immersive VR environment **(B)** of Zenctuary VR+. Alt text: Two-panel figure illustrating the Zenctuary VR+ system. Panel A shows the operator-facing interface used to supervise and control VR sessions. Panel B shows an example of the nature-based immersive virtual environment presented to participants.

Each session is planned to last approximately 15–20 min and will begin with a brief standardized orientation to the available navigation and interaction options. This duration is a guideline rather than a required exposure dose. Participants may pause or discontinue any session at any time. The intervention program comprises six scheduled sessions, with full program completion defined as participation in all six. No minimum within-session duration or number of interactions is required. Sessions terminated by the operator for safety or clinical reasons will be recorded as early terminations. After each session, the operator will complete the standardized closure, post-session interview, and session documentation procedures described in Section 2.7.2.

The main design features of Zenctuary VR+ and their hypothesized experiential and psychological targets are summarized in Table 2. These mappings represent theoretical design assumptions rather than evidence that any individual component causes a specific psychological mechanism. Agency-related, perceived control, engagement, and mastery-like experiences will be explored qualitatively to contextualize changes in psychological distress and other outcomes.

**Table 2:** Zenctuary VR+ components and hypothesized experiential and psychological targets.

Intervention component	Immediate experience	Hypothesized experiential/psychological target
Nature-based immersive environments	Calm, pleasant, restorative context	Emotional regulation and positive affect
Gaze-assisted teleportation and navigational choices	Choosing where to go	Perceived control and decision-making
Low-cognitive-load interaction design	Reduced likelihood of failure or frustration	Engagement and willingness to continue
Optional object interaction and goal-directed activities	Acting on the environment and seeing a result	Agency and action–outcome contingency
Repeated self-initiated actions	Successfully influencing the virtual environment	Mastery-like experiences and self-efficacy
Operator-assisted accessibility support	Supported participation despite limitations	Inclusive participation while preserving autonomy

### 2.9 Control

Participants allocated to the control group will receive a passive immersive VR experience matched to the active condition in hardware, virtual environments, session frequency and duration, standardized onboarding, operator supervision, and safety monitoring.

The control condition will be delivered using the same Meta Quest 3 head-mounted display and will take place within the same nature-based virtual environments used in the active intervention. However, interactive elements, task-related activities, and participant-controlled environmental interactions will be disabled. Participants will remain seated on a virtual bench and observe the virtual environment without engaging in goal-directed activities.

An immersive passive control condition was chosen to isolate the potential contribution of interaction-related mechanisms from the general effects of VR exposure and immersive nature-based content (26, 27). For this purpose, a dedicated “Bench mode” configuration was created in the operator application. In this mode, participants observe the same virtual environments without interaction points, navigation, or goal-directed tasks. Both study groups will therefore receive comparable exposure to immersive nature-based content, while differing primarily in the availability of agency-supporting and control-related interaction opportunities.

This design allows the study to examine whether the hypothesized benefits of the intervention are attributable primarily to the mechanism-oriented interactive components of Zenctuary VR+, rather than to immersion in a virtual natural environment alone.

### 2.10 Concomitant care and clinical management

All participants will continue to receive standard medical care throughout the study. Participation in the trial will not replace, delay, or restrict any diagnostic, therapeutic, rehabilitation, psychological, nursing, or medical procedure considered necessary by the treating clinical team.

Clinical care will remain under the responsibility of the treating physicians and clinical staff at each participating site. Any clinically relevant change in the participant's condition that may affect safe participation will be considered when deciding whether to proceed with, postpone, or discontinue a VR session.

No usual care will be withheld from either study group. The active VR intervention and passive VR control condition will be delivered as additional study procedures alongside routine inpatient care.

### 2.11 Safety monitoring and adverse events

Although the Zenctuary VR+ intervention is considered low risk, the study population consists of hospitalized older adults with chronic medical conditions or other conditions requiring complex inpatient care who may be vulnerable to fatigue, dizziness, discomfort, or balance-related incidents. Therefore, safety monitoring procedures will be implemented throughout the study. All VR sessions will be delivered in a seated or semi-seated position under the supervision of trained study personnel.

Before each VR session, the operator will briefly assess whether the participant is in an appropriate condition to take part in the session. Temporary reasons for postponing or cancelling a session may include acute illness, severe fatigue, severe pain, acute visual disturbance, nausea, dizziness, reduced alertness, or any other condition judged by the clinical team or operator to make participation unsafe on that day.

No additional standardized cybersickness questionnaire will be administered in order to minimize assessment burden in this hospitalized older adult population. Instead, safety and tolerability will be monitored through direct observation, participant self-report during and immediately after the session, and a standardized session log completed by the operator.

Potential adverse events include cybersickness-related symptoms, such as nausea, dizziness, disorientation, eyestrain, headache, fatigue, or transient discomfort, as well as emotional distress, anxiety, loss of balance, pain, or any unexpected physical or psychological reaction occurring during or immediately after VR exposure. Previous studies suggest that immersive VR is generally well tolerated by older adults when appropriate supervision and usability adaptations are provided, although monitoring of cybersickness and tolerability remains recommended (18–21).

Participants will be informed before each session that they may pause or discontinue the session at any time without providing a reason and without any consequences for their medical care or continued participation in the study. The operator will continuously monitor participants during VR exposure and will terminate the session immediately if the participant reports significant discomfort, severe cybersickness symptoms, acute emotional distress, pain, loss of balance or risk of falling, or requests discontinuation.

If a participant experiences any clinically relevant adverse reaction, including acute emotional distress, marked anxiety, disorientation, severe cybersickness symptoms, pain, loss of balance, or risk of falling, the operator will immediately stop the VR exposure and remove the head-mounted display. The participant will be reoriented to the physical environment and supported in a seated or otherwise safe position. The operator will provide calm reassurance and assess whether symptoms resolve after stopping the session. If symptoms persist, are clinically significant, or raise concern for participant safety, the responsible ward nurse, physician, psychologist, or other appropriate clinical staff will be notified according to local institutional procedures.

All session interruptions, operator assistance, early terminations, adverse events, emotional distress reactions, and tolerability issues will be documented in the study log. Documentation will include the nature and type of event, timing, severity, duration, assistance provided, action taken, outcome, and the suspected relationship to the intervention. Serious adverse events will be reported to the responsible ethics committee and participating institution according to applicable regulations and institutional requirements.

Safety and tolerability will be summarized descriptively as part of the study analysis, including the number and type of adverse events, early session terminations, missed sessions due to temporary clinical condition, withdrawals related to discomfort, and any events judged to be related to VR exposure.

### 2.12 Statistical analysis

The statistical analysis plan was developed to evaluate the effectiveness of the Zenctuary VR+ intervention on the primary outcome, psychological distress as measured by the HADS total score. The primary analysis will evaluate the overall group × time interaction in HADS total score across the three predefined assessment occasions (T0, T1, and T2). Within this model, the prespecified principal treatment contrast will assess the between-group difference in change from baseline (T0) to post-intervention (T1), representing the immediate post-intervention effect. The corresponding T0–T2 contrast will be reported as a prespecified follow-up comparison to assess the persistence of any intervention effect.

All statistical tests will be two-sided, and statistical significance will be set at *α* = 0.05. Analyses will primarily follow the intention-to-treat principle, with participants analyzed according to their randomized group allocation. Secondary outcomes and mechanism-related analyses, including learned helplessness-related constructs, will be interpreted as exploratory. Statistical analyses will be conducted using R or SPSS.

#### 2.12.1 Sample size considerations

The sample size calculation was based on the primary outcome, the total score of the Hospital Anxiety and Depression Scale (HADS). As no previous randomized controlled trial has evaluated the Zenctuary VR+ intervention or a directly comparable interactive vs. passive immersive VR intervention in hospitalized older adults, the calculation was based on a conservative small-to-moderate expected effect for the group × time interaction.

Using G*Power 3.1.9.7 (42) for an *a priori* repeated-measures within–between interaction analysis, the sample-size calculation was based on the overall group×time interaction across the three planned measurement occasions. The calculation assumed an effect size of *f* = 0.15, a significance level of *α* = 0.05, statistical power of 0.80, two study groups, three repeated measurement occasions, a correlation of 0.50 among repeated measures, and a nonsphericity correction of *ε* = 0.80. Under these assumptions, the minimum required total sample size was 86 participants (actual power = 0.804). To account for potential attrition, incomplete follow-up, or missing questionnaire data in this hospitalized older adult population, the planned sample size was increased to 100 participants, with 50 participants allocated to the active VR intervention group and 50 participants allocated to the passive VR control group. This corresponds to an allowance of approximately 14% for attrition, incomplete follow-up, or missing outcome data.

Analyses of secondary outcomes, mediation pathways, and learned helplessness-related constructs will be considered exploratory.

#### 2.12.2 Data analyses

Descriptive statistics will be used to summarize baseline demographic and clinical characteristics of the study population. Continuous variables will be reported as means and standard deviations or medians and interquartile ranges, depending on distribution. Categorical variables will be reported as frequencies and percentages.

The primary outcome, HADS total score, will be analyzed using a linear mixed model (43). The model will include group, time, study site, and the group×time interaction as fixed effects, with participant included as a random effect to account for repeated measurements within individuals. Study site is included to account for the site-stratified randomization. Time will be treated as a categorical variable corresponding to the predefined assessment points: baseline (T0), post-intervention (T1), and four-week follow-up (T2). The passive VR control group and baseline assessment will serve as reference categories.

The primary analysis will test the overall group × time interaction across the three assessment occasions. To provide a clinically interpretable estimate of the immediate intervention effect, the prespecified principal treatment contrast will compare the between-group difference in change from T0 to T1. The corresponding T0–T2 contrast will be reported as a prespecified follow-up comparison to evaluate the persistence of any intervention effect. Estimated marginal means, between-group differences in change, 95% confidence intervals, and effect sizes will be reported.

Secondary continuous outcomes, including self-esteem, self-efficacy, hopelessness, depressive symptoms, and learned helplessness-related measures, will be analyzed using similar mixed-model approaches where repeated measurements are available. These analyses will be considered secondary and exploratory. The HADS anxiety and depression subscales will be analyzed separately as exploratory outcomes to characterize symptom-specific patterns of change. GDS scores will be analyzed as a secondary measure of depressive symptoms and interpreted in relation to, but separately from, the HADS depression subscale.

Missing data patterns will be examined and reported. Linear mixed models will use all available repeated-measures data and are expected to provide valid estimates under the assumption that data are missing at random. If the extent or pattern of missing data suggests potential bias, additional sensitivity analyses may be performed, including complete-case analyses and, if appropriate, multiple imputation.

Exploratory correlation analyses will be conducted to examine associations between changes in psychological distress and changes in learned helplessness-related constructs, including self-efficacy, self-esteem, hopelessness, and LHS scores. Exploratory mediation analyses may be conducted to examine whether changes in self-efficacy, self-esteem, or hopelessness are statistically associated with changes in psychological distress in a manner consistent with the hypothesized mechanism. Because potential mediators and HADS are assessed over the same main study intervals, these analyses will not be interpreted as establishing temporal or causal mediation. These analyses will be interpreted cautiously due to the exploratory nature of the mediation hypotheses and the expected sample size.

LHS scores will be analyzed as exploratory mechanism-related outcomes. Given the absence of a fully validated Hungarian version at the time of the trial, findings involving the LHS will be interpreted cautiously and will not be used as primary evidence of intervention effectiveness. Any formal psychometric evaluation of the Hungarian LHS, including reliability, factor structure, and validity analyses, will be conducted and reported separately from the primary trial effectiveness analysis.

Qualitative data will be analyzed using thematic analysis (44). Analysis will be conducted primarily on verbatim transcripts of audio-recorded post-session and final interviews. Structured written interview summaries from non-recorded sessions and operator observational notes will be analyzed as supplementary contextual material and will remain distinguishable from verbatim interview data throughout the analytic process.

The analysis will involve familiarization with the data, initial coding, development and refinement of candidate themes, and interpretation of themes in relation to participant experience, emotional responses, perceived benefits, difficulties, acceptability, engagement, perceived control, observer vs. active-participant experience, barriers to use, and perceived changes across sessions. Exploratory attention will also be paid to agency-related narrative patterns, including references to choice, self-initiated action, successful action, passivity, limitation, help-seeking, and future-oriented intentions.

Reflexivity will be addressed by documenting the analytic decisions and the researchers' roles in data collection and interpretation. Particular attention will be given to the potential influence of the dual role of session operators as both intervention facilitators and interviewers. Qualitative findings will be used to contextualize and help interpret the quantitative trial outcomes rather than as formal quantitative measures of agency, perceived control, or intervention effectiveness. Integration will occur at the interpretation stage by comparing qualitative patterns with the direction and magnitude of quantitative outcome changes.

No task-completion or minimum-exposure threshold will be used to define adherence. Intervention exposure will be characterized descriptively using the number of sessions attended, session duration, operator-recorded stations visited and their order, occurrence of participant-initiated navigation or interaction, predefined interactive activities engaged with, assistance required, and early session termination. These session-level measures will be summarized by randomized group to characterize the actual behavioral separation between the active and passive conditions. Within the active intervention group, exploratory analyses will examine associations between interaction exposure and change in HADS total score from T0 to T1. These analyses will be interpreted as exploratory exposure–response associations rather than causal estimates of treatment efficacy. Full program completion will be defined as participation in all six scheduled sessions. Reasons for missed sessions or discontinuation will be categorized as intervention-related, such as lack of interest, discomfort, or perceived difficulty, or unrelated to intervention acceptability, such as discharge, transfer, or change in medical condition. Safety-related events will be summarized by type, severity, timing, and relationship to the intervention.

Access to the final dataset will be restricted to authorized members of the research team. Data sharing procedures are described in the Data Availability Statement.

### 2.13 Data management

All study data will be collected and managed in accordance with applicable data protection regulations and institutional requirements. Participants will be assigned a unique study identification code, and all research data will be recorded using this code rather than directly identifying personal information.

Data will be entered into a secure, password-protected research database or an equivalent approved research data management system. Directly identifiable information, including participant names and contact details, will be stored separately from research data in a protected identification file accessible only to authorized members of the study team.

Questionnaire data, cognitive screening results, baseline demographic and clinical data, intervention attendance, session logs, adverse events, and follow-up information will be recorded in pseudonymized form. Access to the study database will be restricted to designated members of the research team according to role and responsibility. Data entry and data quality procedures will be implemented to minimize errors, including consistency checks and review of missing or implausible values.

Any paper-based study documents, including signed informed consent forms, will be stored securely at the participating study sites in locked storage facilities. Digital data will be stored on secure institutional servers or approved research data management platforms. Data transfer between study sites and the coordinating center will be performed using secure, approved channels.

The linkage between participant identity and study identification code will be maintained separately and will not be included in the analysis dataset. The final analysis dataset will contain pseudonymized data only. Study data will be retained for the period required by institutional, ethical, and legal regulations and will then be archived or destroyed according to applicable procedures.

Only aggregated and anonymized results will be reported in publications and conference presentations. No individual participant will be identifiable in any study output.

No biological specimens will be collected as part of this trial. Data collection will consist of questionnaire data, screening results, intervention attendance records, session logs, adverse event logs, qualitative post-session notes, and follow-up information. Where audio recordings are made, files will be labeled only with the participant's study identification code and session number, stored securely, and accessed only by authorized members of the research team for qualitative documentation and analysis.

### 2.14 Trial oversight, monitoring, and protocol amendments

The study will be coordinated by the research team at Moholy-Nagy University of Art and Design Budapest in collaboration with the participating clinical centers. Trial oversight will include regular coordination meetings, review of recruitment progress, review of session completion and adherence, monitoring of adverse event logs, and documentation of protocol deviations.

Given the low-risk nature of the intervention and the absence of medicinal product testing, no independent data monitoring committee is planned. Safety monitoring will be performed continuously by trained study personnel during VR sessions and reviewed by the study coordination team. No interim efficacy analysis is planned.

Substantial protocol amendments will be submitted to the relevant ethics and regulatory authorities for approval before implementation, where required. Approved amendments will be communicated to participating sites and study personnel, and the trial registry will be updated as appropriate. Minor administrative changes that do not affect participant safety, study objectives, eligibility criteria, intervention procedures, or outcome assessment will be documented by the study coordination team.

### 2.15 Trial status and ethical aspects

The study has received national ethical and regulatory approval in Hungary. The research was authorized by the National Centre for Public Health and Pharmacy (NNGYK; authorization number: NNGYK/27626-8/2026; research registration number: 1175), based on the expert ethical opinion of the Scientific and Research Ethics Committee of the Medical Research Council (ETT TUKEB; reference number: BM/12784-1/2026).

The trial is currently in the preparation phase. Participant recruitment is planned to begin after completion of site-level preparations. The trial was registered at ClinicalTrials.gov (NCT07712549) before enrollment of the first participant.

Written informed consent will be obtained from all participants before any study-specific procedures are initiated. Participation will be voluntary, and participants will be informed that they may withdraw from the study at any time without any consequences for their medical care.

The study will be conducted in accordance with the principles of the Declaration of Helsinki, applicable Hungarian regulations, institutional requirements, and relevant data protection legislation. All study data will be handled in pseudonymized form, and only aggregated, anonymized results will be reported in publications and conference presentations.

The study team will monitor participant safety throughout the study period. Any serious adverse events or protocol-relevant safety issues will be reported according to institutional and regulatory requirements.

## 3 Discussion

This study protocol describes a multicenter randomized controlled trial designed to evaluate the effectiveness of Zenctuary VR+, a mechanism-oriented, nature-based immersive virtual reality intervention for hospitalized older adults. The trial will compare an active interactive VR condition with a passive VR control condition matched for hardware, visual environment, exposure schedule, and operator supervision. The primary aim is to determine whether the active intervention reduces psychological distress, as measured by the HADS total score, compared with passive exposure to the same virtual environments.

The study is grounded in the theoretical assumption that prolonged hospitalization may create conditions that increase vulnerability to helplessness-related psychological processes in some older adults. These conditions may include reduced autonomy, limited opportunities for self-directed activity, externally regulated routines, and fewer visible links between personal action and environmental or care-related outcomes. Zenctuary VR+ was designed to address these vulnerability conditions by offering repeated opportunities for safe, low-demand interaction, environmental influence, choice, successful action, and meaningful engagement.

Zenctuary VR+ differs from passive nature-based VR by providing opportunities for self-initiated interaction and visible action–outcome contingency. These experiences are hypothesized to support perceived control, agency-related experience, and self-efficacy; however, the present trial is designed primarily to test comparative effectiveness, and these proposed mechanisms will be explored rather than formally established.

Another strength is the focus on a clinically relevant and underrepresented population. Hospitalized older adults with chronic medical conditions or other conditions requiring complex inpatient care may experience limited mobility, reduced autonomy, and psychological distress, yet they are often excluded from technology-based intervention studies because of concerns regarding frailty, usability, cognitive burden, and safety. The present trial addresses these concerns through seated delivery, low-cognitive-load interaction design, operator-assisted accessibility, standardized safety monitoring, and flexible assessment procedures to reduce participant burden.

The study also includes secondary and exploratory outcomes related to self-efficacy, self-esteem, hopelessness, depressive symptoms, and learned helplessness, allowing examination of whether changes in psychological distress are accompanied by changes in these broader psychological constructs.

Several limitations should be acknowledged. Due to the nature of the intervention, participants and operators cannot be blinded to group allocation. Although outcome assessors will remain blinded, accidental disclosure of allocation cannot be fully excluded. The study population is expected to be clinically heterogeneous, as participants will be recruited from different inpatient settings, which may introduce variability in baseline functioning, medical condition, and rehabilitation context. Because eligibility does not require elevated baseline psychological distress, floor effects may limit the magnitude of detectable improvement among participants with low baseline HADS scores. Although patient-reported assessment is appropriate for psychological distress, reliance on self-report may introduce response or expectancy effects, particularly because participants cannot be blinded to intervention allocation. In addition, discharge, transfer, or changes in medical condition may result in missed sessions or follow-up assessments; these reasons will be documented separately from intervention-related discontinuation.

The conservative psychiatric exclusion criteria may also limit the generalizability of the findings to older inpatients with a history of psychotic disorders or those receiving antipsychotic medication. Moreover, secondary mechanism-related analyses, including mediation analyses, will be exploratory and may be limited by sample size.

A further methodological consideration concerns the assessment of learned helplessness. As a fully validated Hungarian version of the LHS is not currently available, LHS-related findings from the present trial will be interpreted as exploratory. The cultural adaptation and psychometric evaluation of the Hungarian LHS will be reported separately and will not form part of the primary effectiveness conclusions of this randomized controlled trial.

The multicomponent nature of Zenctuary VR+ limits attribution of any observed effect to a single feature. The active condition combines several interrelated interactive features, including navigation, object interaction, goal-directed activities, choice, and environmental influence, within a standardized intervention context that includes onboarding and operator support in both groups. Accordingly, any between-group difference will be interpreted as the added effect of the interactive feature package as a whole, rather than evidence that any individual feature is causally responsible or that agency, perceived control, or another proposed mechanism mediates the effect. Operator supervision is provided in both groups, and onboarding, assistance, and safety procedures are standardized across sites to reduce contextual differences. Because engagement with the interactive components is optional, actual interaction exposure may vary substantially within the active condition. Operator-recorded session-level interaction measures will therefore be used to contextualize intervention fidelity and exploratory mechanism-related findings.

Despite these limitations, the trial has the potential to contribute to the development of theoretically grounded, clinically feasible VR interventions for older adults in hospital settings. The study is designed to determine whether interactive Zenctuary VR+ reduces psychological distress to a greater extent than passive exposure to the same virtual environments. It is not designed to establish superiority over usual care. The qualitative and exploratory mechanism-related analyses may nevertheless help contextualize the quantitative findings and inform future studies specifically designed to test these mechanisms. If the intervention shows benefit relative to the passive VR control condition, further research will be required to evaluate its effectiveness in broader clinical populations, implementation requirements, and scalability in routine care.

## 4 Ethics and dissemination

Any study-related harm or clinically relevant adverse reaction will be managed according to local institutional procedures and reported according to applicable regulatory and ethical requirements. Participants will continue to have access to standard clinical care during and after study participation. Compensation and liability coverage will follow the requirements applicable to approved medical research in Hungary and the participating institutions.

The results of the trial will be disseminated regardless of the direction or statistical significance of the findings. Dissemination will include peer-reviewed journal publications, conference presentations, trial registry updates, and project-related communication activities. Authorship will follow standard scientific authorship criteria based on substantial contributions to the conception, design, implementation, analysis, interpretation, and reporting of the study.

The study does not involve the collection of biological specimens. Separate consent may be requested for follow-up contact after discharge when telephone-based follow-up assessment is required.

## Data Availability

No participant-level trial data were analyzed for the present study protocol. The individual participant data to be collected during the trial will include sensitive health-related information from hospitalized older adults. In accordance with the data-sharing plan registered at ClinicalTrials.gov (NCT07712549), participant-level data will not be shared with researchers outside the study consortium under the current arrangements. Study data will be securely stored and accessible only to authorized research personnel. The IPD Sharing Plan is currently registered as “Undecided”. The possibility of future data sharing may be reconsidered following completion of the trial, subject to applicable data protection regulations, ethical approvals, participant consent provisions, and institutional policies.
