# Supplementary material for: A mechanism-oriented immersive virtual reality intervention for psychological distress and helplessness-related processes in hospitalized older adults: study protocol for a multicenter randomized controlled trial
Source: Front Med Technol. 2026 Oct 26;8:1940940. doi: 10.3389/fmedt.2026.1940940 (PMC13745731; doi:10.3389/fmedt.2026.1940940)
Supplement: Supplementary file 1 [file Table1.docx]

Supplementary Material

1. **Supplementary Table 1. SPIRIT 2025 checklist mapping for the ZVR+ trial protocol**

| **Section / topic** | **Item** | **SPIRIT 2025 checklist item** | **Location in ZVR+ manuscript v4.5** | **Status / notes** |
| --- | --- | --- | --- | --- |
| Administrative information | 1a | Title stating the trial design, population, and interventions, with identification as a protocol | Title page | Addressed |
| Administrative information | 1b | Structured summary of trial design and methods, including WHO Trial Registration Data Set items | Abstract; protocol identification block | Addressed |
| Administrative information | 2 | Protocol version date and identifier | Title page | Addressed |
| Roles and responsibilities | 3a | Names, affiliations, and roles of protocol contributors | Title page; 5 Author contributions | Addressed |
| Roles and responsibilities | 3b | Name and contact information for the trial sponsor | 6 Funding; correspondence details; title page | Addressed |
| Roles and responsibilities | 3c | Role of trial sponsor and funders in design, conduct, analysis, and reporting | 6 Funding | Addressed |
| Roles and responsibilities | 3d | Composition, roles, and responsibilities of coordinating site, committees, data management team, and oversight groups, if applicable | 2.14 Trial Oversight, Monitoring, and Protocol Amendments | Addressed |
| Open science | 4 | Trial registry name, identifying number, URL, and date of registration; if not registered, intended registry | Title page; Abstract; 2.15 Trial Status and Ethical Aspects | Addressed |
| Open science | 5 | Where the trial protocol and statistical analysis plan can be accessed | 2.12 Statistical Analysis; protocol publication; 9 Data Availability Statement | Addressed The manuscript itself serves as the protocol publication and includes the statistical analysis plan. |
| Open science | 6 | Where and how de-identified participant data, code, and materials will be accessible | 9 Data Availability Statement; 2.13 Data management | Addressed |
| Open science | 7a | Sources of funding and other support | 6 Funding | Addressed |
| Open science | 7b | Financial and other conflicts of interest for investigators and key committee members | 8 Conflict of interest | Addressed |
| Open science | 8 | Plans to communicate trial results to participants, healthcare professionals, the public, and other relevant groups; authorship policy | Abstract; 4 Ethics and Dissemination; 5 Author contributions | Addressed |
| Introduction | 9a | Scientific background and rationale, including relevant benefits and harms for each intervention | 1 Introduction; Figure 1; 2.11 Safety Monitoring and Adverse Events | Addressed |
| Introduction | 9b | Explanation for choice of comparator | 1 Introduction; 2.9 Control | Addressed |
| Introduction | 10 | Specific objectives related to benefits and harms | Abstract Objective; end of Introduction; 2.11 Safety Monitoring | Addressed |
| Methods: PPI and trial design | 11 | Patient and public involvement in design, conduct, and reporting | 2.1.3 Patient and Public Involvement | Addressed |
| Methods: PPI and trial design | 12 | Description of trial design including type, allocation ratio, and framework | 2.1 Study Design | Addressed |
| Methods: participants, interventions, outcomes | 13 | Trial settings and locations | 2.1.1 Study Setting; title page affiliations | Addressed |
| Methods: participants, interventions, outcomes | 14a | Eligibility criteria for participants | 2.2 Participants and eligibility criteria; 2.2.1 Inclusion criteria; 2.2.2 Exclusion criteria | Addressed |
| Methods: participants, interventions, outcomes | 14b | Eligibility criteria for sites and individuals delivering interventions, if applicable | 2.1.1 Study Setting; 2.4 Training | Addressed |
| Methods: participants, interventions, outcomes | 15a | Intervention and comparator details sufficient for replication, including how, when, and by whom administered | 2.8 Study intervention; 2.9 Control; Table 2; Figure 3 | Addressed |
| Methods: participants, interventions, outcomes | 15b | Criteria for discontinuing or modifying allocated intervention/comparator for a participant | 2.10 Concomitant Care; 2.11 Safety Monitoring and Adverse Events | Addressed |
| Methods: participants, interventions, outcomes | 15c | Strategies to improve adherence and monitor intervention/comparator fidelity/adherence | 2.4 Training; 2.7.2 Secondary Outcomes; Table 1; 2.12.2 Data Analyses | Addressed |
| Methods: participants, interventions, outcomes | 15d | Concomitant care permitted or prohibited during the trial | 2.10 Concomitant Care and Clinical Management | Addressed |
| Methods: participants, interventions, outcomes | 16 | Primary and secondary outcomes with variable, analysis metric, aggregation, and time point | 2.7.1 Primary Outcome; 2.7.2 Secondary Outcomes; Table 1; 2.12.2 Data Analyses | Addressed |
| Methods: participants, interventions, outcomes | 17 | How harms are defined and assessed | 2.11 Safety Monitoring and Adverse Events; Table 1; 2.12.2 Data Analyses | Addressed |
| Methods: participants, interventions, outcomes | 18 | Participant timeline of enrolment, interventions, assessments, and visits; schematic diagram recommended | Figure 2; Table 1; 2.7.2 Secondary Outcomes | Addressed |
| Methods: participants, interventions, outcomes | 19 | Sample size determination and assumptions | 2.12.1 Sample Size Considerations | Addressed |
| Methods: participants, interventions, outcomes | 20 | Recruitment strategies to achieve target sample size | 2.3 Recruitment | Addressed |
| Methods: assignment of interventions | 21a | Who generates random allocation sequence and method used | 2.5 Randomization and Allocation Concealment | Addressed |
| Methods: assignment of interventions | 21b | Type of randomization and stratification factors | 2.5 Randomization and Allocation Concealment | Addressed |
| Methods: assignment of interventions | 22 | Allocation concealment mechanism | 2.5 Randomization and Allocation Concealment | Addressed |
| Methods: assignment of interventions | 23 | Implementation: who enrolls/assigns and access to allocation sequence | 2.5 Randomization and Allocation Concealment | Addressed |
| Methods: assignment of interventions | 24a | Who will be blinded after assignment | 2.1 Study Design; 2.6 Blinding | Addressed |
| Methods: assignment of interventions | 24b | How blinding will be achieved and similarity of interventions, if blinded | 2.6 Blinding; 2.9 Control | Addressed |
| Methods: assignment of interventions | 24c | Unblinding circumstances and procedures, if blinded | 2.6 Blinding | Addressed |
| Methods: data collection, management, analysis | 25a | Plans for assessment and data collection, data quality processes, instruments and validity | 2.4 Training; 2.7 Measurements and Assessments; 2.7.1; 2.7.2; Table 1 | Addressed |
| Methods: data collection, management, analysis | 25b | Plans for participant retention and complete follow-up; outcome data after discontinuation/deviation | 2.3 Recruitment; 2.7 Measurements and Assessments; Table 1 | Addressed |
| Methods: data collection, management, analysis | 26 | Data entry, coding, security, storage, and quality processes | 2.13 Data management | Addressed |
| Methods: data collection, management, analysis | 27a | Statistical methods for primary/secondary outcomes including harms | 2.12 Statistical Analysis; 2.12.2 Data Analyses; 2.11 Safety Monitoring | Addressed |
| Methods: data collection, management, analysis | 27b | Definition of who is included in each analysis and in which group | 2.12 Statistical Analysis | Addressed |
| Methods: data collection, management, analysis | 27c | How missing data will be handled | 2.12.2 Data Analyses | Addressed |
| Methods: data collection, management, analysis | 27d | Methods for additional analyses such as subgroup/sensitivity analyses | 2.12.2 Data Analyses | Addressed |
| Methods: monitoring | 28a | Data monitoring committee composition/role or explanation why not needed | 2.14 Trial Oversight, Monitoring, and Protocol Amendments | Addressed |
| Methods: monitoring | 28b | Interim analyses and stopping guidelines | 2.14 Trial Oversight; 2.11 Safety Monitoring | Addressed |
| Methods: monitoring | 29 | Frequency and procedures for monitoring trial conduct | 2.14 Trial Oversight; 2.11 Safety Monitoring | Addressed |
| Ethics | 30 | Plans for research ethics committee/institutional review board approval | 2.15 Trial Status and Ethical Aspects; Ethics Statement | Addressed |
| Ethics | 31 | Plans for communicating important protocol modifications | 2.14 Trial Oversight, Monitoring, and Protocol Amendments | Addressed |
| Ethics | 32a | Who obtains informed consent/assent and how | 2.3 Recruitment; 2.15 Trial Status and Ethical Aspects; Ethics Statement | Addressed |
| Ethics | 32b | Additional consent for participant data/specimens in ancillary studies, if applicable | 2.3 Recruitment; 4 Ethics and Dissemination; 2.13 Data management | Addressed |
| Ethics | 33 | Confidentiality of personal information before, during, and after trial | 2.13 Data management; 2.15 Trial Status and Ethical Aspects; 9 Data Availability Statement | Addressed |
| Ethics | 34 | Ancillary/post-trial care and compensation for trial-related harm | 4 Ethics and Dissemination; 2.10 Concomitant Care | Addressed |
